# Cip2a/miR-301a feedback loop promotes cell proliferation and invasion of triple-negative breast cancer

**DOI:** 10.7150/jca.35704

**Published:** 2019-10-15

**Authors:** Jiang Yin, Danyang Chen, Kai Luo, Minying Lu, Yixue Gu, Shanshan Zeng, Xiangzhou Chen, Ying Song, Zhijie Zhang, Guopei Zheng, Zhimin He, Hao Liu

**Affiliations:** Guangzhou Key Laboratory of "Translational Medicine on Malignant Tumor Treatment, Affiliated Cancer Hospital and Institute of Guangzhou Medical University, Guangzhou, 510095, PR China.

**Keywords:** Cip2a, miR-301a, triple-negative breast cancer, cell proliferation, cell invasion

## Abstract

Triple-negative breast cancer (TNBC) is a highly aggressive breast cancer subtype and lacks effective targeted therapies. Cancerous inhibitor of protein phosphatase 2A (Cip2a) is an oncogene that is known to inhibit PP2A tumor suppressor activity in human malignancies. We previously demonstrated that Cip2a is a novel target for the treatment of TNBC. However, the functional roles of Cip2a in TNBC progression are still not fully characterized. In this study, we identified that miR-301a is a novel target of Cip2a in TNBC cell lines by miRNA microarray analysis. We found that Cip2a increases E2F1 expression, which in turn transcriptional activates miR-301a by occupying the miR-301a host gene SKA2 promoter. Moreover, we found that miR-301a level is significantly increased in TNBC tissues, and up-regulation of miR-301a is responsible for Cip2a-induced cell proliferation and invasion of TNBC cells. Furthermore, miR-301a feedback promotes the expression of Cip2a via activation of ERK/CREB signaling. Together, our study suggests an auto-regulatory feedback loop between Cip2a and miR-301a and this auto-regulatory loop might play an important role in TNBC progression.

## Introduction

Breast cancer is the most frequently diagnosed cancer and the leading cause of cancer death in women^1^. Breast cancer is a heterogeneous disease in terms of tumor histology, clinical presentation, and response to therapy. There are four major subtypes based on molecular markers ER/PR and Her2 status: luminal A, luminal B, HER2 positive, and triple-negative breast cancer (TNBC)^2^. TNBC is characterized by the lack of expression of estrogen and progesterone receptors and an absence of HER2 amplification, accounts for 15-20% of all diagnosed breast cancers^3^. TNBC is a highly aggressive breast cancer subtype and lacks effective targeted therapies^4^. Thus, understanding of the molecular mechanisms and identifying biological markers of TNBC progression is urgently required to help provide more effective treatments against this disease.

Cancerous inhibitor of protein phosphatase 2A (Cip2a) is an oncogene that is known to inhibit PP2A tumor suppressor activity in human malignancies^5^. The aberrant expression of Cip2a has been observed in different types of cancer cells. In breast cancer, Cip2a has been shown associated with clinical aggressivity and promotes the malignant growth^6^. More important, analysis of Cip2a expression in human breast tumors revealed a trend toward high Cip2a expression in TNBC cases compared with luminal and HER2^+^ tumors^7^, ^8^, ^9^. Accordingly, Cip2a has been reported to determine the drug sensitivity to TNBC cells via regulating PP2A-p-AKT signaling^10^, ^11^, which suggested that targeting Cip2a is a novel approach for the treatment of TNBC. However, the functional roles of Cip2a in TNBC progression are still not fully characterized.

In the past decade, a class of small noncoding RNAs known as microRNAs (miRNAs) bind target mRNAs at complementary sites in their 3'-untranslated regions (3'-UTRs), thereby suppressing the expression of the target gene at the post-transcriptional level^12^. MiRNAs has emerged as a major regulator of the initiation and progression of human cancers, including breast cancer^13^, ^14^. Therefore, a promising approach to elucidate the molecular issues involved in the acquisition of an enhanced aggressive phenotype essential for Cip2a-related tumor progression of TNBC is the investigation of dysregulated miRNAs.

In this study, we identified that miR-301a is a novel target of Cip2a by miRNA microarray analysis. We found that Cip2a increases E2F1 expression, which in turn transcriptional activates miR-301a by occupying the miR-301a host gene SKA2 promoter. Moreover, we demonstrated that increased miR-301a expression is responsible for Cip2a-induced cell proliferation and invasion of TNBC cells. Furthermore, miR-301a activates ERK/CREB signaling, results in an increase the expression of Cip2a. Altogether, these results suggest an auto-regulatory feedback loop between Cip2a and miR-301a and this auto-regulatory loop might play an important role in TNBC progression.

## Materials and Methods

### Cell lines and cell culture

The human breast cancer cell line MDA-MB-231 and BT549 were obtained from the American Type Culture Collection. The cells were cultured in Dulbecco's Modified Eagle Medium (Invitrogen, Carlsbad, CA) containing 10% fetal calf serum (Invitrogen), 100 IU/ml penicillin (Sigma, St. Louis, MO), and 100 μg/ml streptomycin (Sigma) in a humidified incubator of 5% CO_2_ at 37 °C. Cell lines were tested for authenticity using short tandem repeats (STR) genotyping.

### Clinical sample

Thirty-one of fresh primary breast cancer tissues and matched adjacent breast tissues used in this study were collected from the Affiliated Tumor Hospital of Guangzhou Medical University. Written informed consent was obtained from all study participants. This study was approved by the Ethics Committee of Guangzhou Medical University Authority. The collection and use of tissues followed the procedures that are in accordance with the ethical standards as formulated in the Helsinki Declaration.

### MiRNA microarray analysis

Total RNA, containing miRNA, was extracted from tissue samples using TRIzol reagent (Invitrogen, Carlsbad, CA) according to the manufacturer's instructions. The miRNA expression profiles were generated by using the Affymetrix GeneChip miRNA Array v. 4.0 (Affymetrix). Briefly, the flashTag Biotin RNA Labeling Kit (Affymetrix) was used to label of 1 μg of total RNA, followed by the hybridization overnight according to the manufacturer's instructions. Images were scanned using the GeneChip Scanner 3000 and image analysis was done with the GeneChip Operating Software.

### Cell transfection and virus infection

pCMV6-E2F1, pCMV6-c-myc, and pCMV6-XL5 empty plasmid was purchased from Origene (Rockville, MD). MiR-301a hairpin antagomirs were obtained from GeneCopoeia Inc. (Guangzhou, China). All transfections were conducted using Lipofectamine 2000 according to the manufacturer's instructions (Invitrogen). miR-301a-expressing retroviruses, Cip2a-expressing retroviruses were purchased from GENECHEM (Shanghai, China). The Cip2a short hairpin RNA (shRNA) lentivirus vectors were generated by GeneCopoeia Inc. (Guangzhou, China). The target sequences of Cip2a shRNA was 5'-CUGUGGUUGUGUUUGCACUTT-3'. For selection of TNBC stable cell lines, retroviruses were transduced into BT549 and MDA-MB-231 cells in the presence of polybrene (6 μg/mL, Sigma). Cells were selected with 1 μg/mL puromycin for 7 days.

### Cell proliferation assay

Cells were seeded into 6-well plates and the cell numbers were counted after 0 hour, 24, 48, 72 and 96 h of incubation using a Coulter Counter (Beckman Coulter, Fullerton, CA, USA) in triplicate.

### BrdU cell proliferation assay

Cells were treated with 0.03 mg/ml BrdU for 6-12 h, at 37°C, fixed with 4% paraformaldehyde, washed in 0.1 M PBS (phosphate-buffered saline, pH 7.4) with 1% Triton X-100, and incubated with 1 M Hcl (hydrochloric acid) and 2 M Hcl. A borate buffer (0.1 M) was added and cells were blocked with 5% normal goat serum in 0.1 M PBS in the presence of 1% Triton X-100, 1.0 M glycine. The cells were sequentially incubated with anti-BrdU and secondary antibodies.

### Western blot analysis

Cells were harvested and proteins were extracted using RIPA buffer (50 mM Tris-Cl, pH 8.0, 150 mM NaCl, 5 mM EDTA, 0.1% SDS, 1% NP-40) supplemented with protease inhibitor cocktail. Cell lysates were centrifuged at 12000 rpm for 30 min at 4 °C, supernatants were saved, and protein concentrations were determined by by BCA protein assay (Thermo Scientific, Rockford, Illinois, USA). Protein extracts (40 µg) was resuspended and electrophoresed on 10% sodium dodecyl sulfate polyacrylamide gel and then blotted onto polyvinylidene fluoride membranes (Millipore A) at 200 mA for 1.5 h. Following blocking with 5% nonfat milk in TBST (TBS-1% Tween 20) for 1h, membranes were immunoblotted with primary antibodies overnight at 4 °C and further incubated with secondary horseradish peroxidase-conjugated anti-rabbit. Finally, protein bands were detected by developing the blots with the enhanced chemiluminescence western blot detection kit (Engreen Biosystem, China). Antibodies against β-actin (Cat. #4970), GAPDH (Cat. #3683), p-ERK (Thr202/Tyr204) (Cat. # 4370), p-CREB(Ser133) (Cat. #9198), ERK (Cat. #4695), CREB (Cat. #9197) were from Cell Signaling Technology (Beverly, MA, USA). Antibodies against c-myc (Cat. #sc-40), E2F1 (Cat. #sc-251) were from Santa Cruz Biotechnology (Santa Cruz, CA, USA); Antibodies against SKA2 (Cat. #ab91551), and Cip2a (Cat. #ab99518) were from Abcam (Cambridge, MA, USA).

### RNA Extraction and Quantitative real-time PCR

Total cellular RNA was extracted using TRIzol (Invitrogen, California, USA). For mRNA detection, Cip2a and GAPDH mRNA expression was analyzed by the SYBR Green qRT-PCR according to the manufacturer's instructions (Applied Biosystems). For miRNA detection, the reverse transcribed cDNA was synthesized with the All-in-One™ miRNA First- Strand cDNA Synthesis Kit (GeneCopoeia, Rockville, MD, USA). miR-301a expression was determined with the All-in-One™ miRNA qRT-PCR Detection Kit (GeneCopoeia, Rockville, MD, USA) and U6 snRNA was used as the endogenous control. The following primers were used: GAPDH forward, 5'-GACTCATGACCACAGTCCATGC-3'; GAPDH reverse, 5'-AGAGGCAGGGATGATGTTCTG-3'; Cip2a forward, 5'-ATACTTCAGGACCCACGTTTGAT-3'; Cip2a reverse, 5'-TCTCCAAGTACTAAAGCAGGAAAATCT-3'; miR-301a forward, 5'-GGCAGTGCAATAGTATTGT-3'; miR-301a reverse, 5'-TGGTGTCGTGGAGTCG-3'; U6 forward: 5'-CTCGCTTCGGCAGCACA-3'; U6 reverse: 5'-AACGCTTCACGAATTTGCGT-3'.

### Transwell assay

Cells were seeded onto basement membrane matrix on inserts of a 24-well culture plate (EC matrix, Chemicon, Temecula, CA) and fetal bovine serum was added to the lower chamber as a chemoattractant. After 48 hours, the non-invading cells and EC matrix were gently removed with a cotton swab. The invasive cells located on the lower side of the chamber were stained with Crystal Violet, counted and imaged.

### Wound-healing assay

Cells (5×10^5^) were seeded in six-well plates to grow into a monolayer. After starving in serum free medium for 24 hours, a linear wound was created using a sterile pipette. Photomicrographs were taken by microscopy and the distance migrated was observed at time point 0, 24 hours.

### Chromatin-immunoprecipitation

The ChIP assay was carried out using Millipore EZ-Magna ChIP kit (Millipuro) following the manufacturer's instruction. Briefly, 5 × 10^6^ cells were fixed with 1% formaldehyde and quenched in 0.125 M glycine. Cells were sonicated by Bioruptor Sonication System UCD-300. DNA was immunoprecipitated by either control IgG (Cell Signaling Technology, Cat. #3900), E2F1 (Cell Signaling Technology, Cat. #3742) or CREB (Cell Signaling Technology, Cat. #9197) antibody. Precipitated DNA samples and inputs were amplified by PCR. The primers used for the amplification of E2F1 binding site in SKA2 promoter are:

SKA2 Con Forward primer, 5'-TAAGATGAAGCCAGGCCGAG-3'; Reverse primer, 5'-ACTCCCCCGGCTCTTAGACT-3'; SKA2 Site1 Forward primer, 5'-TGCTAGTACGGCTTGCGG-3'; Reverse primer, 5'-CAACTGGTTTAAAGCGGGCA-3'; SKA2 Site2 Forward primer, 5'-AAGTAGAGAGGAGGGGGCAG-3'; Reverse primer, 5'-ATCTCGCACTCATTGGCTCC-3';

The primers used for the amplification of CREB binding site in Cip2a promoter are: Cip2a Con Forward primer, 5'-TCCCTTGGCCAGATTTTACCT-3'; Reverse primer, 5'-TGCTGCGAGGGAACTGTCTA-3'; Cip2a Site1 Forward primer, 5'-CGCAAATAATAGTGCCATTTTCGTA-3'; Reverse primer, 5'-AGTCATGCCCTGACCAAAGT-3'; Cip2a Site2 Forward primer, 5'-TGCCAATAGGTTGACAATAGGT-3'; Reverse primer, 5'-GAAATGGTCGCTTGCCCCTA-3'.

### Luciferase assays

The SKA2 promoter (-1000 to +1 regions), E2F1 binding site-mutated SKA2 promoter, Cp2a promoter (-1000 to +1 regions), and CREB binding site-mutated Cip2a promoter were inserted into the pGL3-basic vector (Promega, Madison, WI, USA). All constructs were verified by sequencing. For the luciferase reporter assay, cells were co-transfected with pGL3-SKA2 or mut-pGL3-SKA2, and pGL3-Cip2a or mut-pGL3-Cip2a using Lipofectamine 2000. The Renilla luciferase was as internal control. 48 h after transfection, cells were harvested and assayed by the Dual-Luciferase Reporter Assay System (Promega, Madison, WI, USA). The relative firefly luciferase activity was calculated by normalizing transfection efficiency according to the Renilla luciferase activity.

### Statistical analysis

All experiments were performed in triplicate at least. The results of this study are presented as mean ± SD and analyzed by Student's t test with statistical analysis of data using GraphPad Prism 5 (GraphPad Software Inc., San Diego, CA).

## Results

### Cip2a induces miR-301a expression in TNBC cell lines

**Figure 1:**
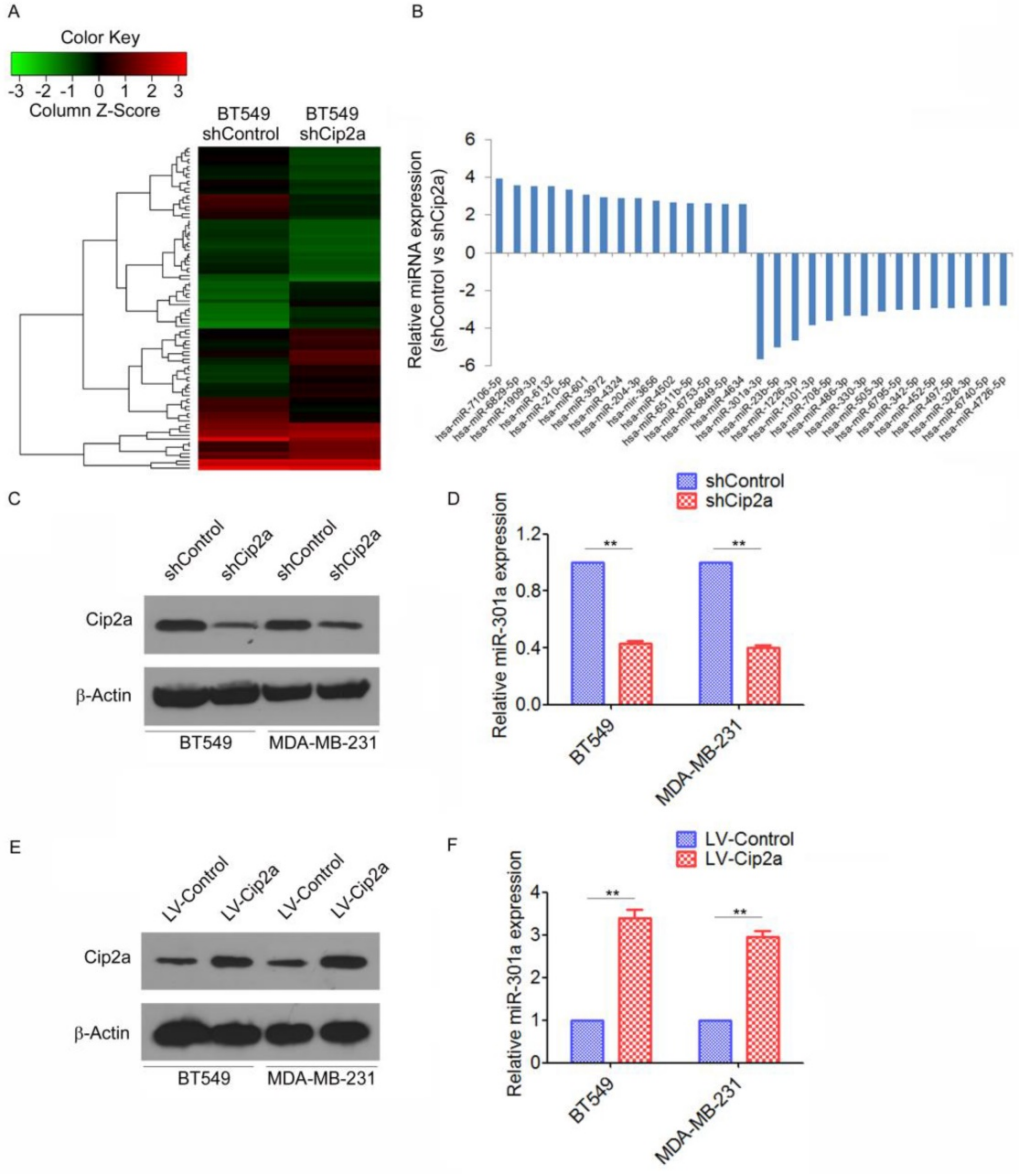
** Cip2a induces miR-301a expression in TNBC cell lines.** (A) Representative heatmaps of differential expression miRNA regulated by Cip2a shRNA. (B) The top 30 differential expression miRNA. (C-D) BT549 and MDA-MB-231 cells were transfected with Cip2a shRNA, the expression of Cip2a was measured by Western blot (C). the expression of miR-301a was measures by qRT-PCR (D). (E-F) BT549 and MDA-MB-231 cells were transfected with Cip2a-expressing vector, the expression of Cip2a was measured by Western blot (E). the expression of miR-301a was measures by qRT-PCR (F). **P <0.01.

As a first step to identify differentially expressed miRNAs regulating by Cip2a in TNBC, we compared the miRNA profiles of the Cip2a-knockdown BT549 cells (BT549-shCip2a) and BT549 transfected with Control shRNA (BT549-shControl) by miRNA microarray analysis. To analyze the microarray data, the expression of each miRNA was normalized to the average median of all the genes and compared between the two cell lines. Using Student's t test, we obtained a list of differentially expressed miRNAs (p ≤ 0.05) in the BT549-shCip2a and BT549-shControl cells. Unsupervised clustering of significantly deregulated miRNAs is presented in Figure 1A. We found that forty-five miRNA were significantly overexpressed (>2-fold) in BT549-shCip2a cells compared with BT549-shControl cells. In addition, forty-four miRNAs were down-regulated (<50%) in BT549-shCip2a cells (Supplementary Table 1). Among them, miR-301a were revealed to be one of the most up-regulated miRNA (Figure 1B). We next validated differential expression of miR-301a by real-time RT-PCR. Normalization of the miRNA expression levels to snRNA RNU6B revealed a significantly decreased in the miR-301a levels in Cip2a-knockdown BT549 or MDA-MB-231 cells compared with control cells, respectively (Fig. 1C, 1D). In contrast, the miR-301a expression levels were significantly increased in Cip2a-overexpressing BT549 or MDA-MB-231 cells compared with control cells (Figure 1E, 1F). Taken together, these data indicated that Cip2a induces miR-301a expression in TBNC cell lines.

### Cip2a induces miR-301a expression through E2F1

**Figure 2:**
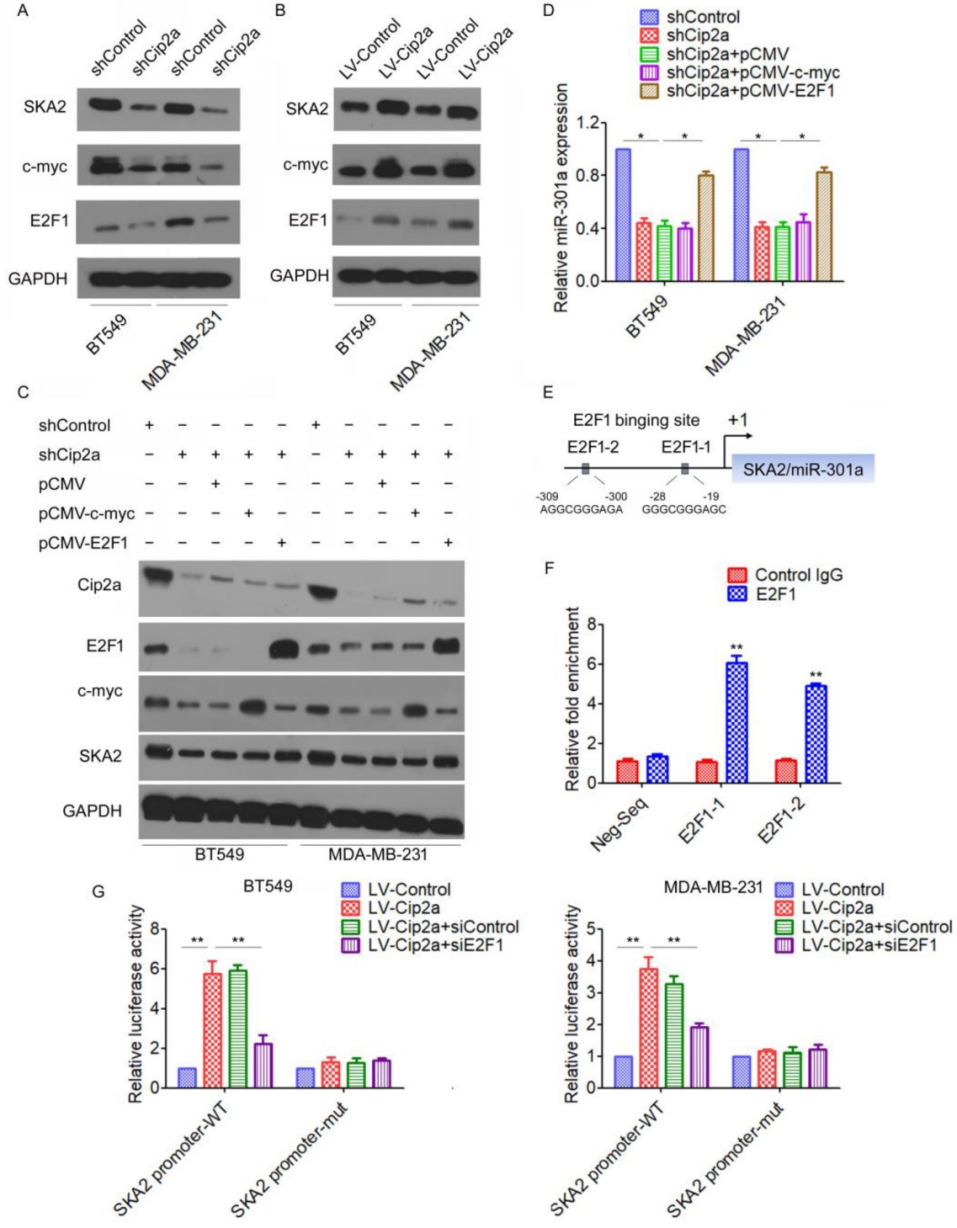
** Cip2a induces miR-301a expression through E2F1.** (A-B) BT549 and MDA-MB-231 cells were transfected with Cip2a shRNA (A) or Cip2a-expressing vector (B), the expression of SKA2, c-myc and E2F1 were measured by Western blot. (C) BT549 and MDA-MB-231 cells were transfected with Cip2a shRNA in combination with pCMV-c-myc, or in combination with pCMV-E2F1, the expression of miR-301a was measures by qRT-PCR. (D) BT549 and MDA-MB-231 cells were transfected with Cip2a shRNA in combination with pCMV-c-myc, or in combination with pCMV-E2F1, the expression of Cip2a, E2F1, c-myc and SKA2 were measured by Western blot. (E) Sequence analysis of the promoter region revealed two conserved E2F1-binding sites at the core promoter region of SKA2/miR-301a (E2F1-1, E2F1-2). (F) The binding of E2F1 on SKA2 promoter region were measure by ChIP analysis. (G) BT549 and MDA-MB-231 cells were transfected with Cip2a-expressing vector in combination with pCMV-E2F1, together with a luciferase reporter construct containing the indicated promoter regions or mutant promoter regions. Relative luciferase activities were measured 48 h after transfection. *P <0.05, **P <0.01.

We next investigated the mechanism by which Cip2a induces miR-301a expression in TNBC cells. miR-301a have been mapped to the intronic regions of SKA2 15. We found that knockdown of Cip2a significantly reduced the expression of SKA2 (Figure 2A), whereas overexpression of Cip2a increased the expression of SKA2 (Figure 2B). Cip2a regulates several transcription factors, including c-myc and E2F1^5^, ^16^, which have been previously implicated in the regulation of miRNA expression^17^, ^18^. Indeed, we confirmed that Cip2a regulates the expression of c-myc and E2F1 transcription factors in TNBC cells (Figure 2A, 2B). To test whether c-myc or E2F1 was able to interfere with the Cip2a-induced upregulation of SKA2 and miR-301a, we analyzed the effect of co-transfection of Cip2a shRNA and c-myc-expressing vector or E2F1-expressing vector on the expression levels of SKA2 and miR-301a. We found that knockdown of Cip2a increased protein levels of SKA2, which was abrogated when Cip2a shRNA was expressed along with E2F1-expressing vector. However, compared to transfection of Cip2a shRNA only, co-transfection of c-myc-expressing vector and Cip2a shRNA only slightly reduced the expression of SKA2 (Figure 2C). Moreover, we found that overexpression of E2F1 reversed that the expression of miR-301a regulating by Cip2a shRNA (Fiugre 2D).

We further elucidated how Cip2a/E2F1 signaling regulates the expression of miR-301a. A sequence analysis of the promoter regions revealed two conserved E2F1-binding sites at the core promoter region of SKA2/miR-301a (E2F1-1, E2F1-2) (Figure 2E). To validate a direct binding of E2F1 to the promoter region, we conducted a ChIP-qPCR assay using anti-E2F1 antibody. The results demonstrated a strong enrichment of E2F1 in the promoter regions of E2F1-1 and E2F1-2 corresponding to the predicted E2F1-binding sites (Figure 2F). To further demonstrate the regulation of Cip2a on the promoter region of SKA2/miR-301a, we performed a luciferase reporter assay. Our results showed that overexpression of Cip2a significantly increased SKA2 promoter activity, whereas transfection of E2F1 siRNA significantly decreased SKA2 promoter activity in Cip2a-overexpressing cells (Figure 2G). Taken together, these studies indicated that Cip2a-induced upregulation of miR-301a is mediated through the regulation of E2F1.

### MiR-301a functions as an oncogene in TNBC

**Figure 3:**
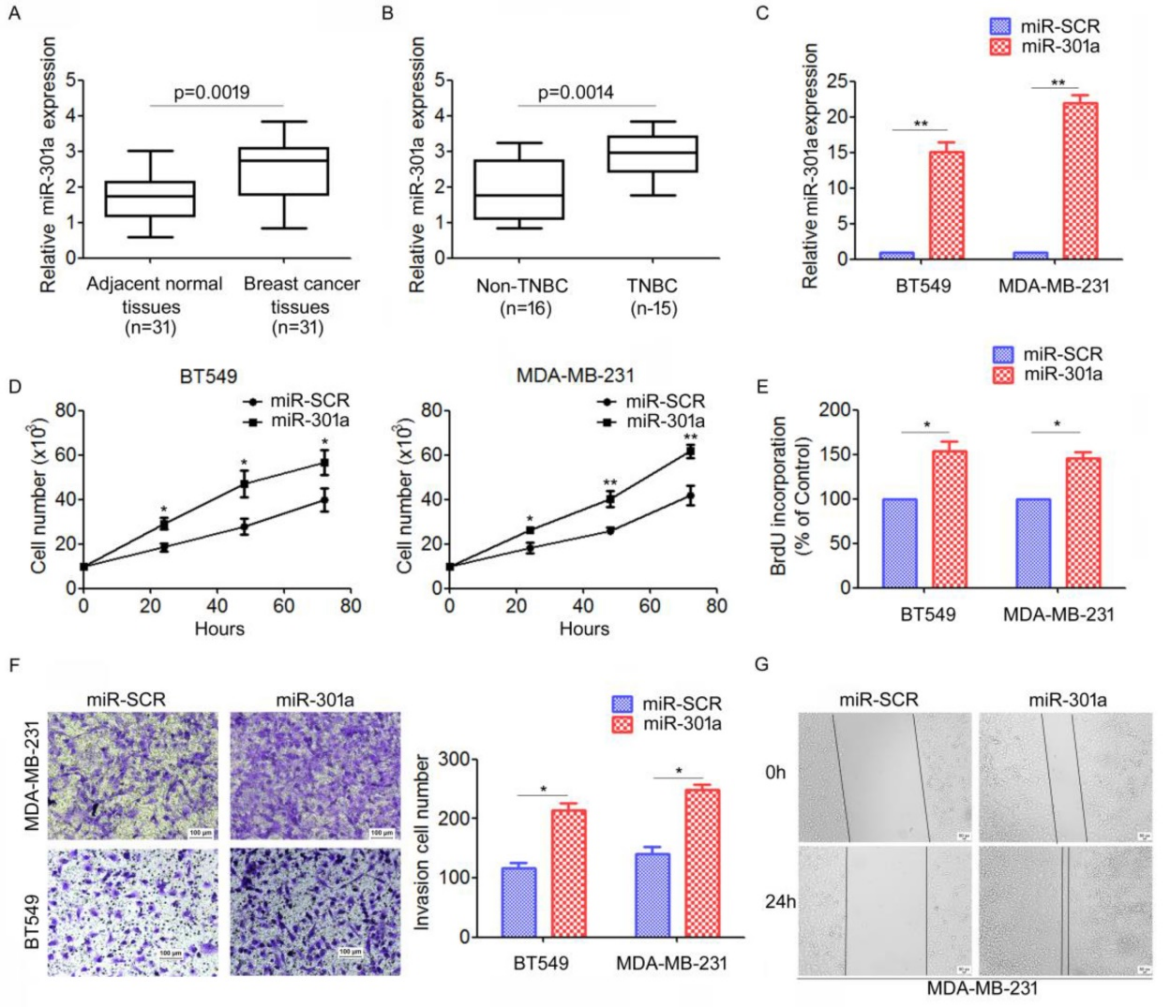
** MiR-301a functions as an oncogene in TNBC.** (A) The levels of miR-30a in 31 paired breast cancer tissues and the corresponding normal adjacent tissues were measures by qRT-PCR. (B) The expression levels of miR-30a in 15 TNBC tissues and 16 non-TNBC (non-triple negative breast cancer) tissues. *P < 0.05. (C) MDA-MB-231 and BT549 cells were infected with miR-30a or miR-SCR lentivirus, the expression levels of miR-30a were evaluated by qRT-PCR. (D-E) The effects of miR-30a on cell proliferation were measured by evaluated by cell counting (D) and BrdU incorporation assay (E). (F-G) The effects of miR-301a on cell migration and invasion were detected using Transwell assays (F) and wound healing assay (G). *P <0.05, **P <0.01.

To investigate the expression patterns of miR-301a in TNBC tissues, qRT-PCR analysis for miR-301a expression were performed on 31 primary tumor samples and matched adjacent breast tissues. We found that miR-301a levels were significantly increased in tumor tissues compared with adjacent tissues (Figure 3A). Moreover, the miR-301a level was significantly increased in TNBC compared with non-triple negative breast cancer (non-TNBC) (Figure 3B).

We further investigated the biological function of miR-301a in TNBC cells. We established stably expressing miR-301a precursor and negative control (miR-SCR) cell lines by lentiviral transfections were established (Figure 3C). The direct cell counting results showed that ectopic expression of miR-301a in BT549 and MDA-MB-231 cells significantly increased cell proliferation compared with the cells expressing miR-SCR (Figure 3D). Using BrdU incorporation assays, we also found that overexpression of miR-301a could increase cell proliferation in BT549 and MDA-MB-231 cells (Figure 3E). Next, we used Transwell and scratch-wound assays to determine the effect on cell migration and invasion. The results showed that ectopic expression of miR-301a significantly promoted cell migration and invasion of BT549 and MDA-MB-231 cells compared with the scrambled controls (Figure 3F, 3G). Taken together, these results indicated that miR-301a has tumor-promoting activity in TNBC.

### Cip2a promotes TNBC cell proliferation and invasion by modulating miR-301a expression

**Figure 4:**
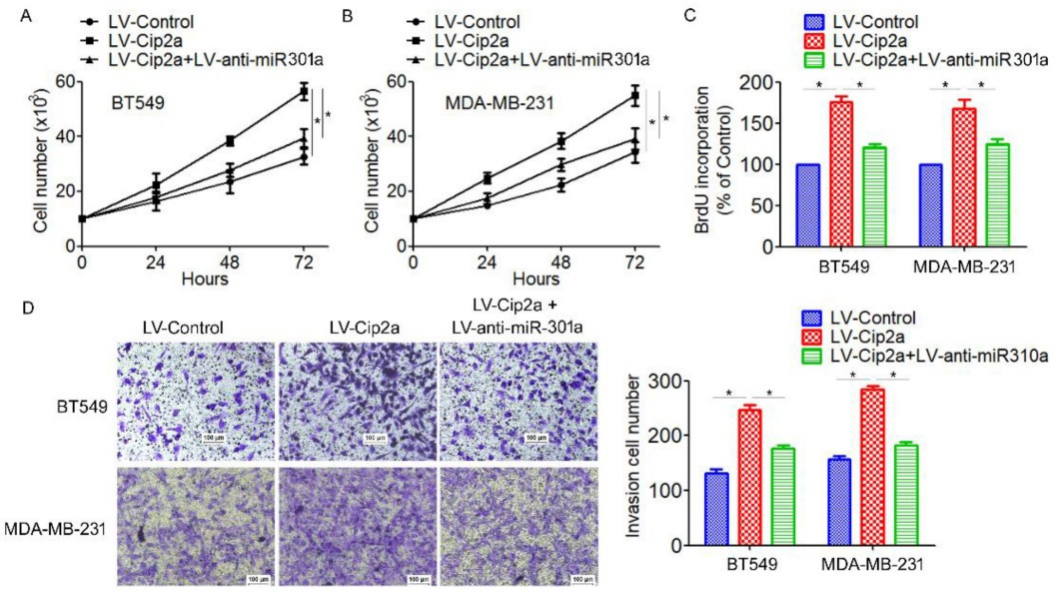
** Cip2a promotes TNBC cell proliferation and invasion by modulating miR-301a expression.** (A) BT549 and (B) MDA-MB-231 cells were transfected with Cip2a-expressing vector alone or co-transfected with anti-miR-301a and Cip2a-epxressing vector, Cell proliferation were measured by evaluated by cell counting. (C) Cell proliferation were measured by evaluated by BrdU incorporation assay. (D) Cell migration and invasion were detected using Transwell assay. *P <0.05.

Based on the above results that miR-301a is a target of Cip2a and its function in TNBC progression, we speculated whether Cip2a promotes TNBC cell proliferation and invasion by modulating miR-301a expression. Cip2a-overexpressing cells were transiently transfected with anti-miR-301a. We found that overexpression of Cip2a significantly increased cell proliferation, whereas co-transfected with Cip2a and anti-miR-301a showed a significant decrease in cell proliferation in BT549 and MDA-MB-231 cells (Figure 4A, 5B). Using BrdU incorporation assays, we also found that inhibition of miR-301a could restore cell proliferation induced by Cip2a (Figure 4C). Moreover, transwell assays showed that inhibition of miR-301a could partially reversed Cip2a-induced cell invasion (Figure 4D). Collectively, these data suggested that miR-301a mediates the promotion effects of Cip2a on TNBC cell proliferation and invasion.

### MiR-301a feedback regulates Cip2a expression by activating ERK/CREB signaling

**Figure 5:**
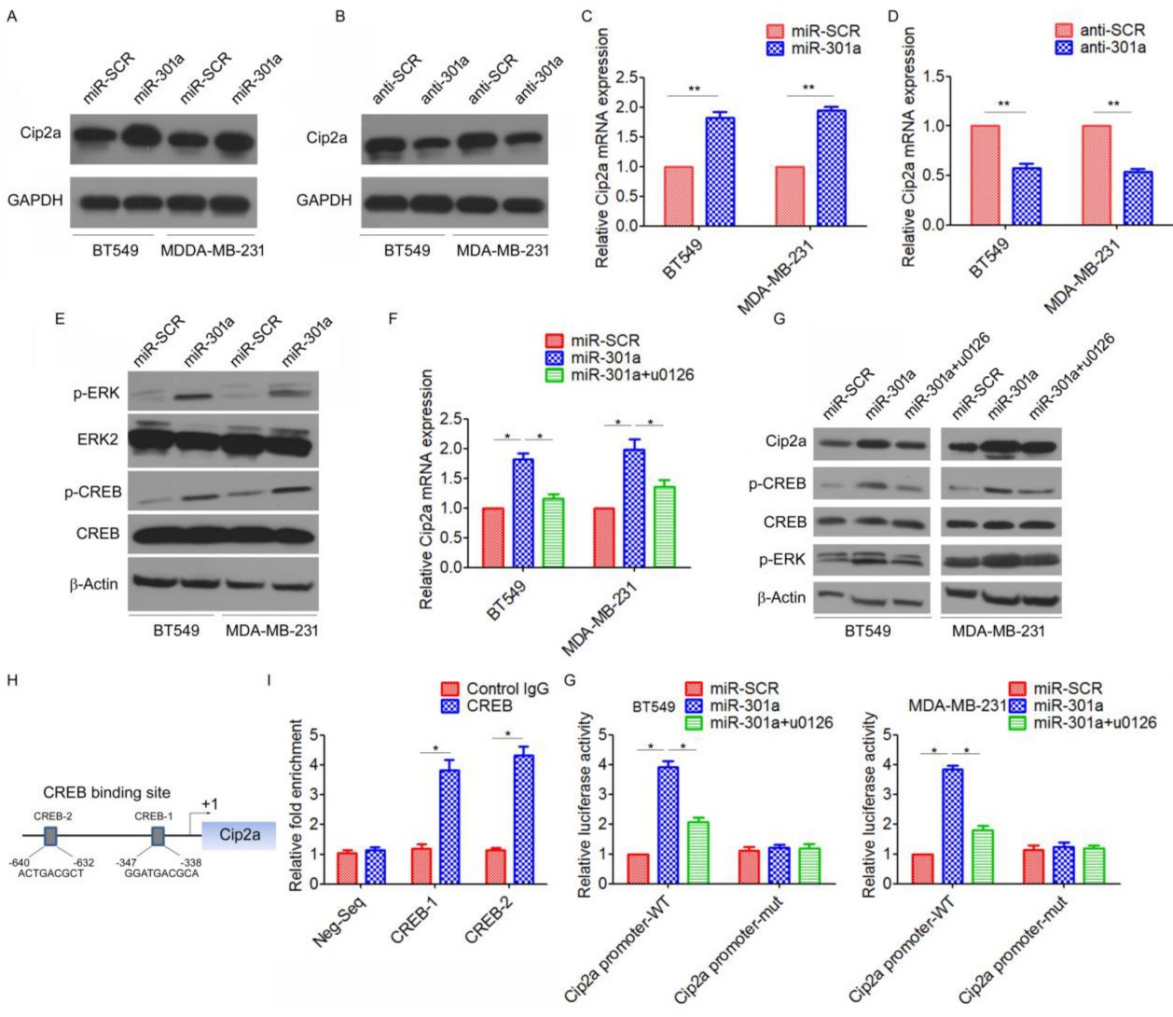
** MiR-301a feedback regulates Cip2a expression by activating ERK/CREB signaling.** (A-B) BT549 and MDA-MB-231 cells were transfected with miR-301a (A) or anti-miR-301a (B), the levels of Cip2a were measured by Western blot. (C-D) BT549 and MDA-MB-231 cells were transfected with miR-301a (C) or anti-miR-301a (D), the mRNA levels of Cip2a were measured by real-time RT-PCR. (E) BT549 and MDA-MB-231 cells were transfected with miR-301a, the phosphorylation levels of Cip2a were measured by Western blot. (F-G) BT549 and MDA-MB-231 cells transfected with miR-301a were treated with ERK inhibitor u0126, (F) the mRNA levels of Cip2a were measured by real-time RT-PCR, (G) the levels of Cip2a, p-ERK and p-CREB were measured by Western blot. (H) Sequence analysis of the promoter region revealed two conserved CEEB-binding sites at the core promoter region of Cip2a (CREB-1, CREB-2). (F) The binding of CREB on Cip2a promoter region were measure by ChIP analysis. (G) BT549 and MDA-MB-231 cells transfected with miR-301a together with a luciferase reporter construct containing the indicated promoter regions or mutant promoter regions, and then treated with ERK inhibitor u0126. Relative luciferase activities were measured 48 h after transfection. Firefly luciferase activity of the reporter construct was normalized to the internal Renilla luciferase activity. *P <0.05, **P <0.01.

Since miRNAs are often components of feedback loops, we hypothesized that miR-301a itself may target components of the Cip2a signaling pathway. Indeed, we found that ectopic expression of miR-301a significantly increased the protein expression of Cip2a, whereas inhibition of miR-301a resulted in the down-regulation of Cip2a (Figure 5A, 5B). qRT-PCR results also demonstrated that overexpression of miR-301a significantly increased Cip2a mRNA expression (Figure 5C, 5D).

We further identified the possible mechanism for the miR-301a-induced upregulation of Cip2a. Previous studies showed that miR-301a activated ERK/CREB pathways by targeting MEOX2 in lung cancer cells^19^. Indeed, we found that ectopic expression of miR-301a significantly increased the phosphorylation levels of ERK and CREB (Figure 5E). Moreover, we found that ERK inhibitor U0126 significantly reversed miR-301a-induced Cip2a expression (Figure 5F, 5G). Furthermore, a sequence analysis of the promoter regions revealed two conserved CREB-binding sites at the core promoter region of Cip2a (CREB-1, CREB-2) (Figure 5H). ChIP-qPCR assay demonstrated a strong enrichment of CREB in the promoter regions of CREB-1 and CREB-2 corresponding to the predicted CREB-binding sites (Figure 5I). To further demonstrate the regulation of miR-301a/ERK/CREB signaling on the promoter activity of Cip2a, we performed a luciferase reporter assay. Our results showed that overexpression of miR-301a significantly increased Cip2a promoter activity, whereas treatment of ERK inhibitor U0126 significantly decreased Cip2a promoter activity in miR-301a-overexpressing cells (Figure 5G). Taken together, these results suggested that miR-301a feedback regulates Cip2a expression by activating ERK/CREB signaling.

MiR-301a has been reported to be dysregulated in a variety of malignant tumors, including breast cancer 29, prostate cancer 30, gastric cancer 31, pancreatic cancer^32^, and colorectal cancer^33^. miRNA-301a was identified as an oncogenic miRNA, which played an important role in promoting cell proliferation and invasion, inhibiting apoptosis and enhancing chemosensitivity both *in vitro* and *in vivo* 15, 34, 35, 36, 37, 38. In this study, we confirmed that MiR-301a functions as an oncogene in TNBC, with the evidence of miR-301a is upregulated in TNBC and ectopic expression of miR-301a significantly promoted cell migration and invasion. In accordance with our results, Yu et al. also demonstrated that miR-301a is upregulated in TNBC cancer tissues compared with adjacent noncancerous tissues, and miR-301a expression was an independent prognostic factor for the survival of patients with TNBC^39^. Moreover, we found that induced by Cip2a inhibition of miR-301a could partially reversed Cip2a-induced cell proliferation and invasion, which indicated that miR-301a mediated the promotion effects of Cip2a on TNBC cell proliferation and invasion.

## Discussion

Because of a lack of targeted therapies (such as hormone therapy or anti-HER2 therapy) for TNBC, discovering the critical molecular mechanisms of TNBC may advance the development of TNBC treatments^20^. In this study, we identified an auto-regulatory feedback loop Cip2a/miR-301a represent a new unifying mechanism of TNBC progression, which might promise to allow multiple therapeutic interventions aimed at blocking its pro-tumor activity.

Cip2a has attracted attention because of its oncogenic potential and tumor-promoting role in several xenograft studies^21^, ^22^, ^23^, ^24^, ^25^, ^26^. The clinical relevance of Cip2a oncoprotein in TNBC aggressiveness has been established^7^, ^27^. We previously demonstrated that high Cip2a expression significantly predicts poor patient survival in TNBC, and Cip2a inhibition significantly impairs cell proliferation and the growth of xenografted tumors in TNBC^8^. Moreover, Cip2a has been shown as a therapeutic determinant mediating the anti-cancer effects of several new agents in TNBC cells, including bortezomib and erlotinib^10^, ^28^. These studies address feasibility of Cip2a targeting as a novel approach to combat TNBC. However, though the oncogenic role of Cip2a in TNBC has been elucidated, the mechanism remains largely unknown.

Using miRNA array analysis, we found that miR-301a is a novel target of Cip2a. Previously studies showed that Cip2a inhibited the tumor suppressor protein PP2A which contributed to consequent effects on the expression of several transcription factors. For example, Cip2a interacted directly with the oncogenic transcription factor MYC, inhibited PP2A activity toward MYC serine 62 (S62), and thereby prevented MYC proteolytic degradation 5. In addition to Myc, Cip2a-mediated inhibition of PP2A is known to stimulate E2F1 pathways^16^. Both c-myc and E2F1 are play an important role in the regulation of miRNA expression. A sequence analysis of miR-301a host gene SKA2 promoter regions contain two conserved E2F1-binding sites. CHIP and luciferase reporter further confirmed that E2F1 could directly bind to SKA2 promoter, and mediate Cip2a-induced upregulation of miR-301a.

One of the most interesting findings in our study was that ectopic miR-301a feedback increased Cip2a expression. A previous study suggested that miR-301a targets MEOX2 transcripts directly to activate ERK/CREB signaling^19^. Indeed, the current study showed that miR-301a increased Cip2a expression by activating ERK/CREB signaling. We found that miR-301a could significantly activate ERK/CREB signaling, which in turn, CREB directly binds to Cip2a promoter and increase its activity. This interesting finding gives strong support that miR-301a and Cip2a are integral part of a regulatory loop in which increasingly high levels of Cip2a promotes cancer progression by up-regulation of miR-301a, whereas high miR-301a expression activates ERK/CREB signaling, which in turn increases the expression of Cip2a.

**Figure 6:**
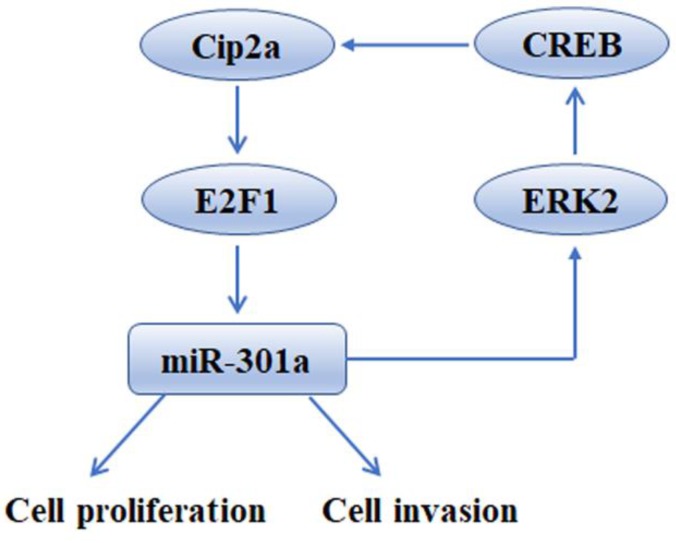
Schematic model of Cip2a/miR-301a feedback loop in promoting cell proliferation and invasion of triple-negative breast cancer.

In summary, our findings shed light on the tumor-promoting function of Cip2a in TNBC, revealing that this promotion effect is mediated, at least partly, through the up-regulation of miR-301a. Moreover, we found that miR-301a feedback up-regulates the expression of Cip2a via activation of ERK/CREB signaling (Figure 6). The uncovered mutual regulation between Cip2a and miR-301a points toward new prognostic and therapeutic options by using components of the Cip2a/E2F1/miR-301a/ERK loop as potential key targets to restrict cancer progression of TNBC.

## Supplementary Material

Supplementary tables.

## Figures and Tables

**Table 1:** Clinicopathological features of BC patients.

Clinicopathological parameters	Number of BCs (%)
**Age**	
< 50	12 (39)
≥ 50	19 (61)
**Histological grade**	
1	9 (29)
2	13 (42)
3	9 (29)
**TNM stage**	
I	6 (19)
II	15 (48)
III-IV	10 (33)
**Lymph node metastasis**	
Negative	23 (74)
Positive	8 (26)
**Molecular subtype**	
ER+	12 (39)
HER2+	4 (13)
TNBC	15 (48)

BC: Breast cancer, TNM: Tumor node and metastasis, ER: Estrogen receptor, HER: Human epidermal growth factor, TNBC: Triple-negative breast cancer
